# Interpreting Estonian Demonstratives: The Effects of Referent’s Distance and Visual Salience

**DOI:** 10.3389/fpsyg.2020.553226

**Published:** 2020-11-20

**Authors:** Maria Reile, Kristiina Averin, Nele Põldver

**Affiliations:** ^1^Institute of Estonian and General Linguistics, University of Tartu, Tartu, Estonia; ^2^Institute of Psychology, University of Tartu, Tartu, Estonia; ^3^Doctoral School of Behavioural, Social and Health Sciences, University of Tartu, Tartu, Estonia

**Keywords:** spatial demonstratives, demonstrative pronouns, demonstrative adverbs, referent distance, visual salience, experimental linguistics, interpretation experiment

## Abstract

Most of the research done with spatial demonstratives (words such as *this*, *here* and *that*, *there*) have focused on the production, not the interpretation, of these words. In addition, emphasis has been largely on demonstrative pronouns, leaving demonstrative adverbs with relatively little research attention. The present study explores the interpretation of both demonstrative pronouns and demonstrative adverbs in Estonian—a Finno-Ugric language with two dialectal-specific demonstrative pronoun systems. In the South-Estonian (SE) dialectal region, two demonstrative pronouns, *see*—“this” and *too—*“that”, are used. In the North-Estonian (NE) region, only one, *see*—“this/that”, is used. The aim of this study is twofold. First, we test if the distance and the visual salience of a referent have an effect on the interpretation of demonstratives. Second, we explore if there is a difference in the interpretation of demonstratives between native speakers from SE and NE. We used an interpretation experiment with 30 participants per group (total *n* = 60) and compared the SE and NE group responses. The results clearly show that the distance of the referent has an effect on how demonstratives are interpreted across the two groups, while the effect of visual salience is inconclusive. There is also a difference in the interpretation of demonstratives between the two dialectal groups. When using the Estonian with an influence of the SE dialect, the NE speakers rely on demonstrative adverbs in interpreting the referential utterance that includes demonstrative pronoun and adverb combinations, whereas the SE speakers also take into account the semantics of demonstrative pronouns. We show that, in addition to an already known difference in the production, there is also a difference in the interpretation of demonstratives between the two groups. In addition, our findings support the recognition that languages that have distance neutral demonstrative pronouns enforce the spatial meaning of a referring utterance by adding demonstrative adverbs. Not only is the interpretation of demonstrative pronouns affected, but the interpretation of demonstrative adverbs as well. The latter shows the importance of studying adverbs also, not just pronouns, and contributes to further knowledge of how demonstratives function.

## Introduction

Demonstratives, such as *this* and *there* in English, are one of the core elements of language as they belong to one of the first words that children acquire (Clark and Sengul, 1978), and they are used to indicate objects in the surroundings of interlocutors (Diessel, 1999, 2013). Diessel (1999) has even proposed that, in every language, there are at least two spatially contrastive demonstratives, demonstrative pronouns, demonstrative adverbs, or demonstrative particles. In this sense, demonstratives can be seen as language universals. However, their functions can differ between languages (e.g., Diessel, 1999; Dixon, 2003), and this makes them an interesting linguistic phenomenon. For instance, in some languages, demonstratives can indicate whether a referent is invisible (as in Khasi language) (Nagaraja, 1985 cited in Diessel, 1999), located down-river or up-river, down-hill or up-hill (as in Dyirbal language) (Dixon, 1972 cited in Diessel, 1999), while in other languages there are no specific demonstratives that would fulfill these functions. Also, in addition to distance indication, demonstratives can be used to express whether the intended referent is in the visual attention of the hearer, such as *şu* in Turkish (Özyürek, 1998; Küntay and Özyürek, 2006), or if the referent is in a joint focus of attention of the interlocutors (Diessel, 2006).

In addition to the different functions that demonstratives can fulfill, there are also different demonstrative pronoun systems. Diessel (1999) classifies demonstrative pronoun systems on the bases of the number of distance contrasts that adnominal demonstratives (demonstrative pronouns with an accompanying noun) make. This means that there can be demonstrative pronoun systems with one-way distance contrast (in these, demonstrative pronouns are distance-neutral, such as in German and in French) and demonstrative pronoun systems with even five distance-contrasts (such as in Koasati) (Diessel, 2013). There is a tendency that the more demonstrative pronouns a system has, the more different aspects of the referent the demonstrative pronouns express.

Empirical research has shown that, in spatial use, demonstratives indicate the distance of the referent from the speaker and the hearer (e.g., Dixon, 2003; Coventry et al., 2008; Diessel, 2013; Levinson, 2018). Moreover, there seems to be a connection between spatial perception and demonstratives as well as memory for object location (Coventry et al., 2008, 2014; Caldano and Coventry, 2019; Gudde et al., 2016). For example, it has been shown that in English and in Spanish the use of so-called distal demonstratives (*that* in English and *aquel* in Spanish) increased when the referent’s distance increased (Coventry et al., 2008). In other words, when the referent was situated in the participant’s extrapersonal space—the space outside one’s grasping distance (di Pellegrino and Làdavas, 2015)—then distal demonstratives were used in referring to that object. Similar results have been found for other languages as well, such as Estonian and Võro language (Reile et al., 2020). In addition, in English, when an object is referred to with a distal demonstrative, then its location is remembered to be more distant than it actually was (Gudde et al., 2016). This highlights the importance of the referent’s distance from the speaker in the choice and use of demonstrative pronouns.

Distance, however, is not the only factor contributing to the choice of demonstratives. Several authors have shown that also the visual salience of the referent or visual access to the referent (Diessel, 1999; Jarbou, 2010; Coventry et al., 2014) affects the use of demonstratives. Joint attention between interlocutors can have an effect on the use (Diessel, 2006) and the interpretation (Stevens and Zhang, 2013) of demonstratives. For example, in English, visually inaccessible objects are referred to with *that* (a distal demonstrative) (Coventry et al., 2014), whereas *this* can be interpreted that the interlocutors share a joint focus of attention (Stevens and Zhang, 2013). Nevertheless, the degree of which these factors influence the use of demonstratives differs between languages. For instance, in Khasi, a Mon Kher language, there is a specific demonstrative to express the invisibility of the referent (Nagaraja, 1985 cited in Diessel, 1999). In other languages, such as English, there are no specific demonstratives for this function, but the use of demonstratives is still influenced by these factors. In Estonian, the visual salience of the referent does not influence the choice of demonstrative pronouns, as in English, but seems to have an effect on how demonstrative adverbs are used (Reile, 2016, 2019). Therefore, the complexity of how different factors actually influence and how they contribute to demonstrative use is not yet fully understood.

While the empirical research on spatial demonstratives has increased, most of these studies have used production experiments to tackle the factors that have an effect on demonstrative use (e.g., Coventry et al., 2008, 2014; Piwek et al., 2008; Peeters et al., 2014; Tóth et al., 2014; Gudde et al., 2016). Nevertheless, there are some studies that focus on the interpretation of demonstrative pronouns (Bonfiglioli et al., 2009; Stevens and Zhang, 2013, 2014; Peeters et al., 2015; Rocca et al., 2020). These studies have shown that distance is not the only factor that can play a role in the interpretation of demonstratives. While distance has been shown to have an effect on the interpretation of demonstratives in several languages, i.e., the incongruent use of demonstratives (using a proximal instead of a distal one in referring to an object outside grasping distance) causes longer reaction times in participants’ responses in Italian, English, and Japanese (Bonfiglioli et al., 2009; Stevens and Zhang, 2013, 2014); other factors, such as shared space of the interlocutors, can override the effects of egocentric distance, such as in Dutch (Peeters et al., 2015). In addition, the effects of distance can be relative in the sense that, when two referents are located in the peripersonal space of the participant, it is not appropriate to refer to both referents with the proximal pronoun (Bonfiglioli et al., 2009), at least in Italian. Even more so, a recent naturalistic fast fMRI experiment in Danish has shown that, while demonstratives are processed in the areas of the brain connected to visuospatial cognition, no statistically significant segregation was found between processing distal and proximal demonstratives (Rocca et al., 2020). Thus, similarly to the production of demonstratives, in the interpretation of demonstratives the effects of distance are also not as straightforward as previously thought.

The current study uses an interpretation experiment to pinpoint the factors that can have an effect on understanding demonstrative meaning in spatial reference, i.e., the use of spatial demonstratives, both demonstrative determiners and demonstrative adverbs, which has been seldom done with this methodological approach. We focus on Estonian which is a Finno-Ugric language that employs at least two demonstrative pronoun systems (Pajusalu, 2009). The use of these systems is related to the historical division of Estonian dialects (Pajusalu et al., 2009). In the North-Estonian (NE) dialectal region, a one-term system is used. This means that the sole demonstrative pronoun that is used is *see*—“this/that”—a distance-neutral demonstrative that refers to any referent regardless of its distance from the speaker. In this one-term demonstrative pronoun system, spatial contrasts are expressed through the use of demonstrative adverbs (see Table 1) (Pajusalu, 2009; Reile, 2015).

**TABLE 1:** Estonian demonstrative pronouns and demonstrative adverbs.

	One-term system (NE)	Two-term system (SE)
Demonstrative pronouns	*see*—“this”/that”	*see*—“this”
		*too—*“that”
Demonstrative adverbs	*siia—*“to here”–*sinna—*“to there”	
	*siin—*“here”–*seal*—“there”	
	*siit—*“from here”–*sealt* from there”	

*NE, North Estonia; SE, South Estonia.*

In the South-Estonian (SE) dialectal region, two demonstrative pronouns *see* and *too* are used. In this demonstrative pronoun system, *see* is the proximal and *too* is the distal demonstrative pronoun. However, *too* has a stronger anchorage to far distance than *see* to near distance (Reile, 2019; Reile et al., 2020). As in the one-term system, demonstrative pronouns can be accompanied by demonstrative adverbs also in the two-term system (see Table 1). While it is not impossible for a distal demonstrative pronoun to be combined with a proximal demonstrative adverb, it is still more common to be combined with a distal demonstrative adverb (Reile, 2016).

Both demonstrative pronouns are used as determiners in both demonstrative pronoun systems. Both demonstrative pronouns are also present in the written language of standard Estonian. However, *see* is far more frequent than *too* (Reile, 2019, p. 29). This suggests that while the Estonian speakers originating from the NE region have an exposure to the demonstrative pronoun *too*, at least in written form, it is highly likely that their interpretation of this demonstrative in spatial reference is different as compared to the Estonian speakers from the SE region.

Previous studies on the production of Estonian demonstratives have shown that, while distance has a straightforward effect on the choice of Estonian demonstratives (Reile et al., 2019, 2020), the effect of visual salience might manifest itself in a more indirect way, that is, rather than influencing the choice between distal or proximal demonstratives, the position of demonstrative adverbs in the word order of a referential utterance is affected (Reile, 2016). In other words, in referring to visually non-salient referents, the distal demonstrative adverb *seal*—“there” precedes the referential noun phrase (NP), and in referring to visually salient referents, the distal demonstrative *seal*—“there” comes after the referential NP (that might also include a demonstrative pronoun). For example, in referring to a visually non-salient book, one might say “*Vaata, seal see/too raamat*!” with a direct translation “Look, there this/that book!”, and for a visually salient book, “*Vaata, see/too raamat seal*!” with a direct translation “Look, this/that book there!” In the current study, we manipulate the word order of the input sentence to put this finding under test.

To find out how participants interpret Estonian demonstrative determiners and adverbs, we conducted an interpretation experiment. The aim was to detect a possible association between the distance of the referent, the visual salience of the referent, and the interpretation of demonstratives. In other words, we were interested in whether some demonstratives in the demonstrative paradigm are preferred more for distant/visually non-salient referents than others.

Considering all the above-mentioned points, we have proposed the following hypotheses:

(1) The distance of the referent has an effect on demonstrative interpretation: when the demonstrative pronoun *too—*“that” or the adverb *sealt—*“(from) there” are heard, a distant referent is chosen, and when the demonstrative pronoun *see*—“this” and the adverb *siit—*“(from) here” are heard, a proximal referent is chosen.
(2) With demonstrative pronoun and adverb combinations, the choice of referent is based on the demonstrative adverbs when the visual scene is incongruent with the possible meaning of a demonstrative pronoun in a heard sentence, i.e., when a distal demonstrative pronoun is heard but the referents are in near space and when a proximal demonstrative pronoun is heard but the referents are in far space.
(3) The visual salience of the referent has an effect on demonstrative interpretation: with demonstrative pronoun and adverb combinations, the visually non-salient referent is chosen when the adverb precedes the pronoun in a heard sentence, and the visually salient referent is chosen when the pronoun precedes the adverb in a heard sentence.
(4) The choices for the referents between the NE and the SE speakers differ when the demonstrative *too* is heard. The SE speakers will show a pattern of choosing the farthest referent of the possible referents, while the NE speakers may choose any referent regardless of their distance.
(5) The reaction times (RTs) for choosing a referent when the demonstrative *too—*“that” is heard (with or without a demonstrative adverb) are slower for the NE speakers than for the SE speakers.

## Materials and Methods

The interpretation experiment consisted of two conditions. First, we tested the effects of the referent’s distance from the speaker on the interpretation of demonstratives. Second, we looked for the effects of the visual salience of the referent on the interpretation of demonstratives.

### Participants

Sixty volunteer participants (mean age, 29.7 years; *SD* = 6.5 years) with normal or corrected-to-normal vision took part in the experiment. Thirty participants originated from the one-way demonstrative system (NE) region and 30 from the two-way system (SE) region. In both groups, there were seven males and 23 females. It was explicitly explained to all the participants that their participation was voluntary and that they could leave the experiment at any time point, and an oral consent for participation was acquired.

### Stimuli and Design

**FIGURE 1:**
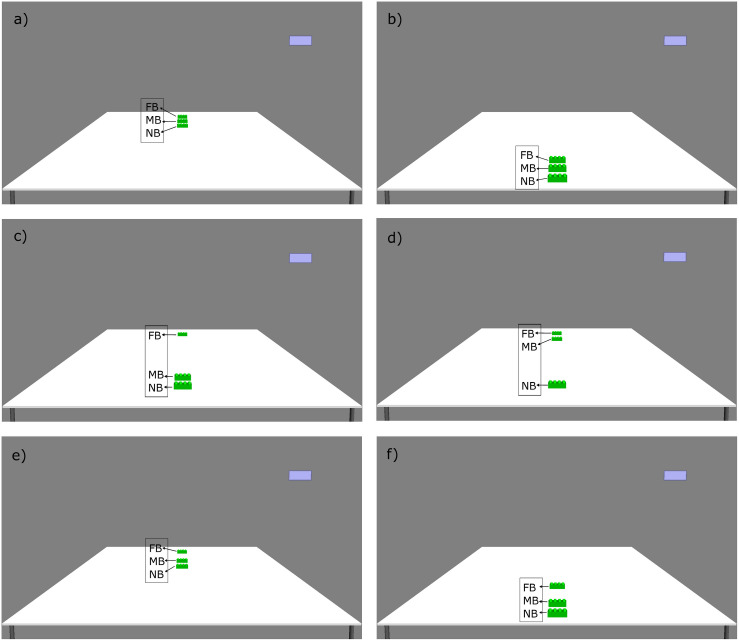
The six picture stimuli used in the distance condition of the experiment, depicting the position of the bricks (green). The blue rectangle in the upper-right corner of the screen marks the response option in case of “do not know.” Note that the text boxes (FB, MB, and NB, referring to the farthest, middle, and nearest brick, respectively) were not presented to the participants on the screen during the experiment. **(a)** Picture 1, **(b)** Picture 2, **(c)** Picture 3, **(d)** Picture 4, **(e)** Picture 5, and **(f)** Picture 6.

**FIGURE 2:**
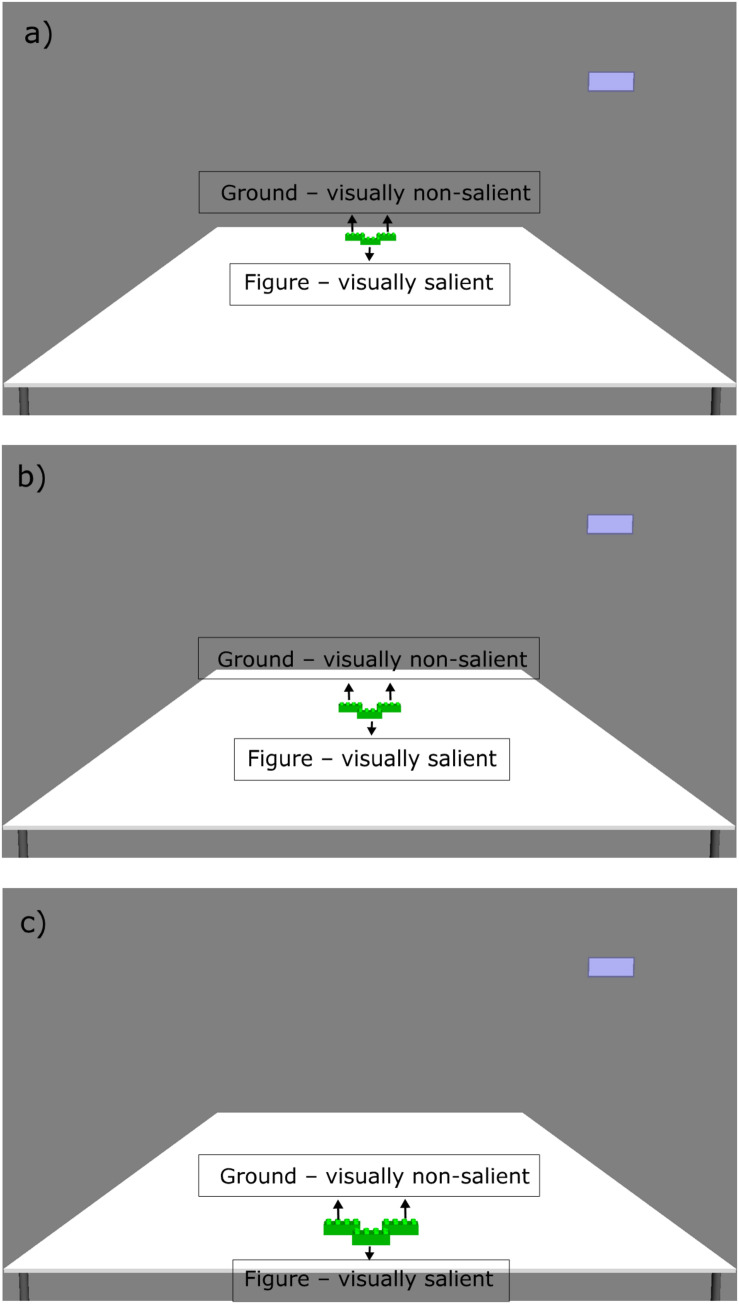
The three picture stimuli used in the visual salience condition of the experiment, depicting the position of the bricks (green). The blue rectangle in the upper-right corner of the screen marks the response option in case of “do not know.” Note that the text boxes (Figure, Ground) were not presented to the participants on the screen during the experiment. **(a)** Picture 7, **(b)** Picture 8, and **(c)** Picture 9.

We used the Psychophysics Toolbox (Brainard, 1997; Pelli, 1997; Kleiner et al., 2007) and its integrated OpenGL commands for Matlab (MathWorks, Natick, MA, United States) to create 3D images (hereafter pictures), run the experiments, and record the data. Every stimulus contained a picture of a table with three green construction bricks (see Figures 1, 2) and a blue rectangle in the upper-right corner. In pictures 1–6, we manipulated the distance of the bricks from the near end of the table. In pictures 7–9, we also manipulated the visual salience of the bricks.

In the distance condition of the experiment, we manipulated the location of referents in six pictures that were designed in a way that proportionally mimicked peri- and extrapersonal space division. The latter has been shown to have an effect on the choice of demonstratives in production experiments (e.g., Coventry et al., 2008, 2014; Reile et al., 2020). This means that, in each of the pictures, the bricks were located either in the supposed peripersonal space of the participant (i.e., near to the participant, henceforth near space) or outside of it (i.e., far from the participant, henceforth far space) (see Figure 1).

In the distance condition of the experiment, the three bricks were positioned on the table in the near space or the far space of the participants. In picture 1, all the three bricks were situated in the near space, that is, at the near the edge of the table. In picture 2, all the bricks were in the far space, which is at the far end of the table. In picture 3, two bricks were in the near space and one in the far space, and in picture 4, two bricks were in the far space and one in the near space. In picture 5, all the bricks were in far space, but one of them was a little farther away than the other two. In picture 6, all the bricks were in near space, but similarly to picture 5, one of the bricks was a little bit farther away than the other two (see Figure 1, pictures 1–6).

In the visual salience condition, we grouped the three referents together to create a figure-ground setting, that is, one in front (visually salient) and two at the back (visually non-salient). In addition to the visual salience, we manipulated the distance of the referent groups. In each picture, the whole brick group was either in the supposed peripersonal space of the participants or outside of it (see Figure 2). In picture 7, the grouped bricks were in far space. In picture 8, the bricks were nearer than in picture 7, but still in far space, and in picture 9, the bricks were placed in near space (see Figure 2, pictures 7–9). Changing the location of the brick group enabled us to test whether the visual salience effect would override the distance effect.

The participants’ task was to look at the picture (one at a time) and choose a brick from the picture by clicking on the brick with a computer mouse. The participants had to make their choice based on the input sentence that they heard from the headphones. When it seemed that the sentence they heard did not match any of the bricks that they saw, they were allowed to click on the blue rectangle on the upper-right corner of the screen, which meant a response “do not know.”

The input sentences that the participants heard were recorded by a female voice and went through an acoustic correction in Praat (Boersma and Weenink, 2007) to exclude the possible effect of intonation on the participants’ choice of the referents. To do that, we overlaid each sentence with a neutral statement intonation contour with downstepped fundamental frequency (F0) peaks on the non-pronominal/content words (declining F0 peaks). The longer sentences (e.g., *võta sealt see klots—*“take from there this brick”) were resynthesized with three F0 peaks, the shorter sentences (*võta see klots—*“take this brick”) included only two F0 peaks. More specifically, we marked the onsets and offsets of every phrase and stressed vowel. F0 at the beginning of the sentence was set at 270 Hz, and at the end of the sentence, it was 190 Hz, regardless of sentence length. The F0 peaks were aligned with one-third of the vowel duration into the vowel. The peak heights from first to the final content word in long sentences were set to 277, 240, and 230 Hz and in short sentences to 270 and 230 Hz. F0 contour between these values was obtained by quadratic interpolation as provided in Praat PSOLA resynthesis method.

The input sentences consisted of either only an adnominal demonstrative pronoun (where a demonstrative pronoun precedes the noun) or combinations of adnominal demonstrative pronouns and demonstrative adverbs. The input sentences were as follows:

(1) *Võta* ***see*** *klots*—“take **this** brick”
(2) *Võta* ***see*** *klots* ***siit***—“take **this** brick from **here**”
(3) *Võta* ***siit see*** *klots*—“take from **here this** brick”
(4) *Võta* ***see*** *klots* ***sealt***—“take **this** brick from **there**”
(5) *Võta* ***sealt see*** *klots*—“take from **there this** brick”
(6) *Võta* ***too*** *klots*—“take **that** brick”
(7) *Võta* ***too*** *klots* ***siit***—“take **that** brick from **here**”
(8) *Võta* ***siit too*** *klots*—“take from **here that** brick”
(9) *Võta* ***too*** *klots* ***sealt***—“take **that** brick from **there**”
(10) *Võta* ***sealt too*** *klots*—“take from **there that** brick”
(11) *Võta väike jänku*—“take the little bunny”

In addition to the input sentences, we also used a filler sentence (no. 11) for control to keep the participants alert throughout the experiment. The filler sentence occurred six times per experiment series.

We used a different order of demonstrative pronouns and demonstrative adverbs in the input sentences because it has been shown that there is a tendency to use demonstrative adverbs in the first position of a referential utterance for visually non-salient referents (Reile, 2016, 2019).

### Procedure

The experiment took place in a semi-darkened room on a Dell Precision M6500 laptop with a screen diameter of 17″ and 1,440 × 900-pixel resolution. The participants were instructed to sit in front of the laptop, put on the headphones, rest their heads on a chinrest in front of them, and hold a computer mouse with their dominant hand.

Before the experiment, the eye-tracking system was calibrated, and the participants were presented with four test-trials. In the test-trials, we did not use any demonstratives but had object descriptive phrases, such as *võta kollane klots*—“take (a) yellow brick.” The eye-tracking measurement data are not analyzed in the scope of the current paper.

The procedure of the experiment was as follows: first, a picture of a table with bricks appeared on the computer screen for 5 s, during which the participants heard the auditory input sentence. After hearing the input sentence, the mouse cursor appeared on the right side of the screen. The participants had to choose one of the three bricks that they thought was the best match to the sentence heard and click on it using the computer mouse. If the participants felt that the input sentence did not apply to any of the bricks, they could click on a blue rectangle on the upper-right corner of the screen, indicating an answer “do not know.” The time starting from the appearance of the cursor until the response (mouse click) was measured in milliseconds (reaction time) and recorded in a text file together with the relevant information per trial: the chosen brick, trial number, condition, and participant ID. After the response was given, a white screen was presented for 1 s, which was thereafter followed by a new trial.

All the pictures and input sentence sequences were blocked and randomized. All the input sentences had three repetitions with each of the pictures (except the filler sentence which was presented six times in each experiment series). Thus, there were 186 (3 × 6 × 10 + 6 controls) trials for the distance series and 96 (3 × 3 × 10 + 6 controls) trials for the visual salience series of the experiment. To minimize the order effect, half of the participants started the experiment with the distance condition and half with the visual salience condition.

### Data Analysis

The data analysis was carried out in R software version 4.0.2 (R Core Team, 2020) using generalized linear mixed effects regression models (GLMM). Similarly to linear mixed models, GLMM allows incorporating fixed and random factors. When fixed factors account for the systematic variability, random factors allow considering the variability from sources other than those in the scope of the present research interest. However, unlike LMM, GLMM does not require the dependent variable to follow a normal distribution (Lo and Andrews, 2015). Thus, for the reaction time analyses, we built general mixed models using the lme4 package (Bates et al., 2015), and for analyzing the choices of the participants, we used the MCMCglmm package that generates GLMM by utilizing Markov chain Monte Carlo and Bayesian methods. This allows specifying a variance structure with prior distributions for fixed and random factors (Hadfield, 2010). The advantages of the package in analyzing choice data include an option to create multinomial models and an ability to deal with issues arising from complete separation. The latter may occur if, in some of the conditions, some levels of the dependent variable have zero choosing frequency (Hadeld, 2012). For response data analysis, whether the participants chose a referent or clicked on the “do not know” rectangle, we built a generalized linear mixed effects regression model using the lme4 package glmer (Bates et al., 2015).

All the models were built separately for distance and visual salience condition. In the models that were based on the RT data and the choice data, the dependent variable was the participants’ reaction time when choosing a brick (for the RT data) or the brick chosen by the participants (for the choice data), respectively. In the distance condition, the dependent variable had three levels: the nearest brick (reference category), the middle brick, and the farthest brick. In the visual salience condition, the dependent variable had two levels: non-salient brick (reference category) and salient brick. The independent variables for both conditions, as well as for both RTs and choice-based models, were as follows: origin of the participants, stimulus picture, and the interaction between the two. Note that, in the visual salience condition, word order was also added as an independent variable. The origin of the participants was a binary variable with the levels NE (reference category) and SE. The stimulus picture had six levels in distance condition, pictures 1 (reference category)–6, and three levels in visual salience condition, pictures 7 (reference category)–9. The word order in visual salience condition had two levels: adverb preceding a pronoun (reference category) and pronoun preceding an adverb. In all the models, the participant’s ID was added as a random effect, and in the RT data-based model, we also included the trial number. From these analyses, the “do not know” rectangle click responses were excluded.

In the models that were based on the response data in distance and visual salience conditions, the dependent variable was the response of the participants, either the “do not know” rectangle click (reference category) or the choice of a brick. The independent variables were the origin of the participant, stimulus picture (the levels are the same as in previous models for both variables), and the input sentence. The input sentence had four levels: *võta see klots siit—*“take this brik from here” (reference category), *võta see klots sealt—*“take this brick from there”, *võta too klots siit—*“take that brick from here”, and *võta too klots sealt—*“take that brick from there”. Note that for a better comparison of the models, we left out the input sentences that included only demonstrative pronouns and merged the input sentences that included the same demonstratives but had a different word order (e.g., *võta see klots siit—*“take this brick from here” was merged with *võta siit see klots—*“take from here this brick”).

## Results

### Distance Condition: Choices of Bricks

We tested hypotheses 1, 2, and 4 using the data from the distance condition. As the aim of hypotheses 2 and 4 was to pinpoint the differences between the two participant groups in the interpretation of specific Estonian demonstratives, we built a separate model for each input sentence (see Table 2).

**TABLE 2:** Results of generalized linear mixed effects regression models predicting the choices of the participants in distance condition.

	*võta see klots siit* “take this brick from here”		*võta too klots siit* “take that brick from here”		*võta see klots sealt* “take this brick from there”		*võta too klots sealt* “take that brick from there”	
				
	Middle	Farthest	Middle	Farthest	Middle	Farthest	Middle	Farthest
Intercept	−3.30***	−3.16***	−1.59***	−1.34***	–0.35	3.25***	0.63	4.22***
Origin SE vs. NE	–0.31	0.11	0.99*	1.71***	1.22*	–0.47	0.42	1.41*
Picture 2 vs. 1	−2.10***	−1.44**	−1.35***	−1.97***	−1.45**	−3.56***	−1.14*	−3.05***
Picture 3 vs. 1	0.15	–0.35	1.13***	−0.63*	0.74	3.57***	1.25	3.39***
Picture 4 vs. 1	−3.11***	−4.26***	−2.51***	−4.32***	2.36***	−2.19***	1.48**	−1.56***
Picture 5 vs. 1	−1.98***	0.08	–0.05	−0.77**	–0.31	–0.73	1.17	1.08*
Picture 6 vs. 1	0.57	1.29***	0.98***	1.00***	1.55	2.84***	1.66	3.13***
Origin SE: picture 2 vs. origin NE: picture 1	NA	NA	NA	NA	–0.34	0.63	NA	NA
Origin SE: picture 3 vs. origin NE: picture 1	NA	NA	NA	NA	2.12*	0.40	NA	NA
Origin SE: picture 4 vs. origin NE: picture 1	NA	NA	NA	NA	−1.33*	0.42	NA	NA
Origin SE: picture 5 vs. origin NE: picture 1	NA	NA	NA	NA	–0.26	0.23	NA	NA
Origin SE: picture 6 vs. origin NE: picture 1	NA	NA	NA	NA	1.42	1.06	NA	NA
Observations	1,662		1,648		1,803		1,806	
Model accuracy (%)	85.61		62.74		76.32		86.82	

The reference category of the dependent variable is the nearest brick. The accuracy of the models was calculated based on the agreement between the bricks actually chosen and the choices suggested by the probabilities predicted by the model. *p < 0.05, **p < 0.01, ***p < 0.001.

**The input sentence *võta see klots siit—*“take this brick from here”**

While the variable origin of the participants had no effect on the choice of the participants, the stimulus picture proved to be statistically significant. Pictures 2 and 4 decreased the likelihood for the participants to choose the middle or the farthest brick over the nearest brick as compared to picture 1 (*p* < 0.001 and *p* < 0.01, respectively, for with picture 2 and *p* < 0.001 and *p* < 0.001, respectively, for with picture 4). Picture 5 decreased the likelihood for the participants to choose the middle brick over the nearest brick (*p* < 0.05) but had no effect on the choice of the farthest brick. Picture 6 increased the likelihood for the participants to choose the farthest brick over the nearest brick (*p* < 0.05) but had no effect on the choice of the middle brick over the nearest brick. There were no statistically significant interactions.

**The input sentence *võta too klots siit*—“take that brick from here”**

Both independent variables origin of the participants and stimulus picture proved to be statistically significant in predicting the choice of the participants. The SE participants were more likely to choose the farthest or the middle brick over the nearest brick (*p* < 0.001 and *p* < 0.01, respectively) than the NE participants. Pictures 2–6 showed a statistically significant effect on the choice of the participants as compared to picture 1. Pictures 2 and 4 decreased the likelihood for the participants to choose the middle or the farthest brick over the nearest brick (all *p* < 0.001). Picture 3 increased the likelihood for the participants to choose the middle brick over the nearest brick (*p* < 0.001) but decreased the likelihood for the participants to choose the farthest brick over the nearest brick (*p* < 0.05). Picture 5 decreased the likelihood for the participants to choose the farthest brick over the nearest brick (*p* < 0.01) but had no effect on the choice of the middle brick. Picture 6 increased the likelihood for the participants to choose the middle or the farthest brick over the nearest brick (both *p* < 0.001). The interaction terms were statistically not significant and thus excluded from the final model.

**The input sentence *võta see klots sealt—*“take this brick from there”**

Both independent variables origin of the participants and stimulus picture proved to be statistically significant in predicting the choices of the participants. The SE participants were more likely to choose the middle brick over the nearest brick (*p* < 0.05) than the NE participants, but there was no effect on the choice of the farthest brick. Pictures 2, 3, 4, and 6 showed a statistically significant effect on the choice of the participants as compared to picture 1. Picture 2 decreased the likelihood of the participants to choose the middle or the farthest brick (*p* < 0.01 and *p* < 0.001, respectively) over the nearest brick. Pictures 3 and 6 increased the likelihood for the participants to choose the farthest brick over the nearest brick (both *p* < 0.001) but had no effect on the choice of the middle brick over the nearest brick. Picture 4 increased the likelihood for the participants to choose the middle brick over the nearest brick (both *p* < 0.001) and decreased the likelihood for the participants to choose the farthest brick over the nearest brick (*p* < 0.001). As for interactions, the interaction between origin of the participants and picture 4 and the origin of the participants and picture 3 proved to be statistically significant. The SE participants were more likely to choose the middle brick (*p* < 0.05) than the NE participants when seeing picture 3 as compared to seeing picture 1. However, when seeing picture 4 as compared to seeing picture 1, the SE participants were less likely to choose the middle brick (*p* < 0.05) than the NE participants. None of the interactions had an effect in predicting the choice for the farthest brick.

**The input sentence *võta too klots sealt—*“take that brick from there”**

Both independent variables origin of the participants and stimulus picture proved to be statistically significant in predicting the choices of the participants. The SE participants were more likely to choose a farthest brick over the nearest brick (*p* < 0.05) than the NE participants, but there was no effect on the choice of the middle brick over the nearest brick. Pictures 2–6 showed a statistically significant effect on the choice of the participants as compared to picture 1. Picture 2 decreased the likelihood of the participants to choose the middle or the farthest brick over the nearest brick (*p* < 0.05 and *p* < 0.001, respectively). Pictures 3, 5, and 6 increased the likelihood for the participants to choose the farthest brick over the nearest brick (*p* < 0.001, *p* < 0.05, and *p* < 0.001, respectively) but had no effect on the choice of the middle brick. Picture 4 decreased the likelihood for the participants to choose the farthest brick over the nearest brick (*p* < 0.01) and increased the likelihood for the participants to choose the middle brick over the nearest brick (*p* < 0.001). The interaction terms were statistically not significant and thus excluded from the final model.

### Visual Salience Condition: Choices of Bricks

To test hypothesis 3, we used the data from the visual salience condition. As the aim of this hypothesis was to pinpoint the differences in the interpretation of specific Estonian demonstratives in regard to the visual salience of the referents, we built a separate model for each input sentence.

None of the variables included in the models built for the input sentences *võta see klots siit*—“take this brick from here” and *võta too klots siit*—“take that brick from here” had a statistically significant effect on the choices of the participants (see Table 3). The salient brick, which was also the closest one to the participants from the three bricks, was chosen considerably more frequently than the non-salient bricks regardless of which of the pictures were seen or what was the origin of the participants.

**TABLE 3:** Results of generalized linear mixed effects regression models predicting the choices of the participants in the visual salience condition.

	*võta see klots siit* “take this brick from here”	*võta too klots siit* “take that brick from here”	*võta see klots sealt* “take this brick from there”	*võta too klots sealt* “take that brick from there”
Intercept	3.00***	2.38***	0.41	−1.17
Origin SE vs. NE	−1.19	−1.51	−0.32	−1.16
Word order Pron-Adv vs. Adv-Pron	0.21	−0.44	−0.41	−0.29
Picture 8 vs. 7	0.80	0.24	0.18	−0.09
Picture 9 vs. 7	−0.06	−0.63	−2.48***	−2.45**
Origin SE: picture 8 vs. origin NE: picture 7	NA	NA	NA	−0.01
Origin SE: picture 9 vs. origin NE: picture 7	NA	NA	NA	1.94*
Observations	1,662	1,648	1,803	1,806
Model accuracy (%)	92.59	63.30	59.02	72.73

The reference category of the dependent variable is the salient brick. The accuracy of the models was calculated based on the agreement between the bricks actually chosen and the choices suggested by the probabilities predicted by the model. *p < 0.05, **p < 0.01, ***p < 0.001.

**The input sentence *võta see klots sealt—*“take this brick from there”**

The only independent variable that was statistically significant in predicting the choice of the salient brick was the stimulus picture. Picture 9 decreased the likelihood for the participants to choose the salient brick as compared to picture 7 (*p* < 0.001).

**The input sentence *võta too klots sealt—*“take that brick from there”**

There were two statistically significant variables in predicting the choice of the salient brick. First, picture 9 decreased the likelihood for the participants to choose the salient brick as compared to picture 7 (*p* < 0.01). Second, the interaction between origin of the participants and picture 9 increased the likelihood for choosing the salient brick (*p* < 0.05), that is, the SE participants were more likely to choose the salient brick than the NE participants when seeing picture 9 as compared to seeing picture 7.

### Results of the Reaction Time Analyses

Since the aim of hypothesis 5 was to test the differences between the two speaker groups while hearing specific Estonian demonstratives, we built a separate model for each input sentence for both distance and visual salience conditions. None of the independent variables proved to be statistically significant in the models of visual salience condition; therefore, we only present the results of the distance condition (see Table 4).

**TABLE 4:** Results of generalized linear mixed effects models predicting the participants’ reaction times.

	*võta see klots siit* “take	*võta too klots siit* “take	*võta see klots sealt* “take	*võta too klots sealt* “take
	this brick from here”	that brick from here”	this brick from there”	that brick from there”
Intercept	1.39***	1.71***	1.52***	1.62***
Origin SE vs. NE	–0.00	–0.02	0.06	–0.05
Picture 2 vs. 1	0.11	–0.03	0.07	0.02
Picture 3 vs. 1	0.07	–0.13	−0.18***	−0.26***
Picture 4 vs. 1	0.00	−0.28***	0.03	–0.10
Picture 5 vs. 1	0.07	–0.00	–0.10	−0.14**
Picture 6 vs. 1	0.05	–0.05	–0.10	−0.26***
Origin SE: picture 2 vs. origin NE: picture 1	0.00	0.11	NA	NA
Origin SE: picture 3 vs. origin NE: picture 1	–0.07	0.13	NA	NA
Origin SE: picture 4 vs. origin NE: picture 1	−0.15*	0.34***	NA	NA
Origin SE: picture 5 vs. origin NE: picture 1	0.09	0.11	NA	NA
Origin SE: picture 6 vs. origin NE: picture 1	–0.09	0.07	NA	NA
Observations	1,662	1,648	1,803	1,806

*p < 0.05, **p < 0.01, ***p < 0.001.

**The input sentence *võta see klots siit*—“take this brick from here”**

The interaction between origin of the participants and picture 4 decreased (*p* < 0.05) the participants’ RTs. The SE participants were quicker in making a choice than the NE participants while seeing picture 4 as compared to seeing picture 1. No other variables or interactions proved to be statistically significant (all *p*-values exceeded the 0.05 threshold).

**The input sentence *võta too klots siit—*“take that brick from here”**

Picture 4 decreased the RTs significantly as compared to picture 1 (*p* < 0.001) in both participant groups. However, with interaction between origin of the participants and picture 4, the SE participants’ RTs got slower than the NE participants’ RT’s when seeing picture 1 as compared to seeing picture 4. No other variables or interactions proved to be statistically significant (all *p*-values exceeded the 0.05 threshold).

**The input sentence *võta see klots sealt—*“take this brick from there”**

Only stimulus picture 3 had a statistically significant effect on the participants’ RTs. Both participant groups were quicker when they saw picture 3 as compared to picture 1 (*p* < 0.01). This means that making a choice while hearing the input sentences *võta see klots sealt—*“take this brick (from) there” was easier when seeing picture 3 as compared to picture 1. There were no statistically significant interactions between the variables (all *p*-values exceeded the 0.05 threshold).

**The input sentence *võta too klots sealt—*“take that brick from there”**

Stimulus pictures 3, 5, and 6 had a statistically significant effect on the participants’ RTs. Both participant groups were quicker when they saw pictures 3, 5, and 6 as compared to picture 1 (*p* < 0.001, *p* < 0.01, and *p* < 0.001, respectively). This means that making a choice while hearing the input sentences *võta see klots sealt—*“take this brick (from) there” was easier when seeing pictures 3, 5, and 6 as compared to picture 1. There were no statistically significant interactions between the variables (all *p*-values exceeded the 0.05 threshold).

### Responses of the Participants: A Choice for a Brick vs. “Do Not Know”

The results show that there was also a slight difference in the overall responses, whether to choose a brick or opt for the “do not know” rectangle, between the two participant groups. Table 5 presents the results of the response data in the distance and visual salience conditions. Most of the “do not know” responses were for the pictures in which the bricks were all in the same distance, picture 1 and picture 2 (35.2% for NE and 27.9% for SE speakers and 42.9% for NE and 31.9% for SE speakers of the choices, respectively). Thus, for the participants, it was easier to make a choice between the bricks if they were divided between far and near space or if one of the bricks stood out from the rest.

**TABLE 5:** The proportion of “do not know” responses by origin of the participants, distance stimuli, and visual salience stimuli.

	Stimulus picture	NE	SE
		***n*(%)**	***n*(%)**
Distance	Picture 1	254(35.2)	201(27.9)
	Picture 2	309(42.9)	230(31.9)
	Picture 3	70(9.7)	56(7.7)
	Picture 4	57(7.9)	57(7.9)
	Picture 5	180(25.0)	136(18.9)
	Picture 6	108(15.0)	63(8.7)
Visual salience	Picture 7	285(39.6)	152(21.1)
	Picture 8	252(35.0)	87(12.1)
	Picture 9	265(36.8)	115(15.9)

Similarly, the “do not know” answers were frequent in the visual salience condition (see Table 5) (note that the total count of choices is 720 for each picture). Differences in the responses between the SE and the NE groups indicated that the participants could also have a different behavior in their decision to choose or not choose a brick. Therefore, we tested this separately for distance and visual salience conditions.

### Distance Condition: A Choice for a Brick vs. “Do Not Know”

As seen in Table 6, the origin of the participants (NE vs. SE) did not have a statistically significant effect in predicting the participants’ response. However, other independent variables proved to be statistically significant. While pictures 3–6 increased (all *p* < 0.001) the likelihood of the participants to choose a brick rather than clicking on the “do not know” rectangle as compared to picture 1, picture 2 decreased (*p* < 0.001) the same. All input sentences, apart from the input sentence *võta too klots siit—*“take that brick from here,” had an effect on the participants’ responses. The input sentences *võta see klots sealt*—“take this brick from here” and *võta too klots sealt—*“take that brick from there” increased (all *p* < 0.001) the likelihood for the participants to choose a brick when compared to the input sentence *võta see klots siit*—“take this brick from here”.

**TABLE 6:** Results of generalized linear mixed effects models predicting a choice for a brick over “do not know” rectangle clicks in distance and visual salience conditions.

Distance		Visual salience	
	
Independent variables	Brick chosen	Independent variables	Brick chosen
Intercept	1.14	Intercept	0.67
Origin SE vs. NE	1.40	Origin SE vs. NE	2.01*
Picture 2 vs. 1	−0.59***	Picture 8 vs. 7	0.34*
Picture 3 vs. 1	2.49***	Picture 9 vs. 7	0.21
Picture 4 vs. 1	2.79***	ProxPDistA vs. ProxPProxA	0.60***
Picture 5 vs. 1	0.83***	DistPProxA vs. ProxPProxA	−0.29*
Picture 6 vs. 1	1.80***	DistPDistA vs. ProxPProxA	0.48***
ProxPDistA vs. ProxPProxA	0.77***	Origin SE: picture 8 vs. origin NE: picture 7	0.76**
DistPProxA vs. ProxPProxA	–0.07	Origin SE: picture 9 vs. origin NE: picture 7	0.37
DistPDistA vs. ProxPProxA	0.79***		
Origin SE: picture 2 vs. origin NE: picture 1	0.16		
Origin SE: picture 3 vs. origin NE: picture 1	0.12		
Origin SE: picture 4 vs. origin NE: picture 1	–0.21		
Origin SE: picture 5 vs. origin NE: picture 1	0.17		
Origin SE: picture 6 vs. origin NE: picture 1	0.63*		
Observations	8,640		4,320

ProxPDistA, võta see klots sealt—“take this brick from there”; DistPDistA, võta too klots sealt—“take that brick from there”; ProxPProxA, võta see klots siit—“take this brick from here”; DistPProxA, võta too klots siit—“take that brick from here.” *p < 0.05, **p < 0.01, ***p < 0.001.

We also tested for interactions between origin of the participants and stimulus picture (Table 6). All interactions proved to be statistically not significant (all *p* > 0.05) apart from the interaction between the variables origin of the participants and picture 6 (*p* < 0.05). The SE participants were more likely to choose a brick instead of the “do not know” rectangle than the NE participants when seeing picture 6 as compared to seeing picture 1.

### Visual Salience Condition: A Choice for a Brick vs. “Do Not Know”

As seen in Table 6, all independent variables had a statistically significant effect on predicting the participants’ response in visual salience condition. The SE participants were more likely to choose a brick rather than the “do not know” rectangle (*p* < 0.05) when compared to the NE participants. Also, hearing the input sentences *võta see klots sealt—*“take this brick from there” and *võta too klots sealt—*“take that brick from there” increased the likelihood (*p* < 0.001 and *p* < 0.001, respectively) for the participants to choose a brick as compared to hearing the input sentence *võta see klots siit*—“take this brick from here.” However, hearing the input sentence *võta too klots siit—*“take that brick from here” decreased the likelihood (*p* < 0.05) for the participants to choose a brick as compared to hearing the input sentence *võta see klots siit*—“take this brick from here.” As for the effect of stimulus pictures, picture 8 increased the likelihood (*p* < 0.05) for the participants to choose a brick rather than a “do not know” rectangle as compared to picture 7. Picture 9, however, had no effect.

We also tested for the effects of interactions. There was one statistically significant interaction between the variables origin of the participants and picture 8 (*p* < 0.01). The SE participants were more likely to choose a brick rather than the “do not know” rectangle than the NE participants when seeing picture 8 as compared to seeing picture 7.

## Discussion

While the empirical research on demonstratives in spatial use has increased in recent years (e.g., Stevens and Zhang, 2013; Coventry et al., 2014; Peeters et al., 2014; Gudde et al., 2016; Levinson, 2018; Caldano and Coventry, 2019), there are not many studies that concentrate on demonstrative interpretation. Furthermore, most of the studies focus on demonstrative pronouns rather than demonstrative adverbs in well-studied languages, such as English, Dutch, and Japanese. Considering the fact that there are languages that lack the distance contrast on the level of demonstrative pronouns (Diessel, 1999, 2013), it is essential to investigate both demonstrative pronouns and adverbs to gain full understanding on how demonstrative systems work.

This study focuses on the interpretation of Estonian demonstratives—demonstrative determiners and demonstrative adverbs. We conducted an interpretation experiment where we (1) tested for the effects of referent’s distance and visual salience on the interpretation of demonstratives and (2) explored the possible differences in the interpretation of demonstratives between two Estonian native speakers’ groups originating from South Estonia (SE, two-term system users) and North Estonia (NE, one-term system users). Studying two speaker groups, who use different demonstrative pronoun systems, provided us with a better insight on how the demonstrative systems work. We were able to do this since the SE speakers tend to use two demonstrative pronouns, *see*—“this” and *too—*“that,” while the NE speakers use only *see*—“this.” Both speaker groups use all demonstrative adverbs. We will now discuss our findings in the light of each hypothesis.

The results support our first hypothesis, that is, the distance of the referent has an effect on the interpretation of demonstratives: when the demonstrative pronoun *too—*“that” and the adverb *sealt—*“(from) there” are heard, a distant referent is chosen and when the demonstrative pronoun *see*—“this” and the adverb *siit—*“(from) here” are heard, a proximal referent is chosen. This is in line with the previous findings of interpretation (Bonfiglioli et al., 2009) as well as production studies (e.g., Coventry et al., 2008, 2014; Piwek et al., 2008; Tóth et al., 2014; Gudde et al., 2016; Meira and Guirardello-Damian, 2018) in different languages. In our study, the participants tended to choose the farthest referent when hearing input sentences that included the demonstrative pronoun *too—*“that” and the adverb *sealt—*“from there,” and the proximal referent tended to be chosen when hearing the demonstrative pronoun *see*—“this” and the adverb *siit—*“from here.” At the same time, the effect of distance was relative, that is, the participants still chose a referent according to the input sentence heard even if the demonstrative determiner and/or adverb heard was not congruent with the displayed stimulus picture. For example, with picture 6 where the referents were in near space but the last one was a bit farther than the first two, the likelihood for the participants to choose the farthest referent over the nearest one was even higher than with picture 1, where all the referents were in far space. Similar findings on distance relativity have been reported for Italian demonstrative pronouns in a comprehension experiment conducted by Bonfiglioli et al. (2009), where the RTs of the participants were slower if the object that was referred to with a proximal demonstrative pronoun was positioned in the far distance and *vice versa*. This indicates that while a single referent in near space (in peripersonal space) can be referred to with proximal demonstrative, as has been found for several languages (see Coventry et al., 2008, 2014 and Reile et al., 2020), with multiple referents in near space, the distance between the referents themselves will start to influence the interpretation of demonstratives and probably production as well.

The results also support our second hypothesis that, with demonstrative pronoun and adverb combinations, the decision of choosing the referent is based on the demonstrative adverbs when the visual scene is incongruent with a demonstrative pronoun in a sentence heard. However, this hypothesis did not hold true with all the pictures. For example, when the participants heard the input sentence *võta too klots siit—*“take that brick from here” and saw picture 2, where all the referents were in near space, the likelihood for the participants to choose the farthest referent decreased as compared to picture 1, where all the referents were in far space. On the other hand, seeing picture 6, where all the referents were in near space but one was a bit farther than the others, the likelihood for choosing the farthest referent increased as compared to picture 1. This different pattern of choices between picture 2 and picture 6 while hearing the same input sentence suggests that the interpretation of demonstrative pronoun and adverb combinations is more complex than we first predicted. While previous findings with Estonian demonstratives show that demonstrative adverbs have a stronger association with the distance of the referent than demonstrative pronouns (Reile, 2019; Reile et al., 2019), our results suggest that this applies only when the intended referents are all in the same distance with each other. Similar findings to our interpretation study have been found by Meira and Terrill (2005) who argued that, in Lavukaleve language, when the referents are positioned in the same length from each other, these are seen as being in one region, and speakers tend to refer to them with the same demonstrative.

While distance had an effect on demonstrative interpretation across the two participant groups, there was a difference in how they interpreted the meaning of demonstrative determiner and adverb combinations. This brings us to our fourth and fifth hypotheses. The fourth hypothesis was as follows: the choices for the referents between the NE and the SE speakers differ when the demonstrative *too* is heard. The SE speakers will show a pattern of choosing the farthest referent, while the NE speakers may choose any referent regardless of their distance. The results partly support this hypothesis. The origin of the participants proved to make a difference with the input sentences where the distal demonstrative pronoun was combined with a proximal demonstrative adverb and *vice versa* and also when the distal demonstrative pronoun was combined with a distal demonstrative adverb. The SE participants were more likely to choose the farthest or the middle referent when hearing a distal pronoun and proximal adverb combination than the NE participants. With the proximal pronoun and the distal adverb combination, they were more likely to choose the middle referent, and with the distal pronoun and distal adverb combination, they were more likely to choose the farthest referent than the NE participants. This suggests that the SE speakers needed to match the region from where to look for the referent with the demonstrative adverb as well as to decide which of the possible referents in this region would best match the demonstrative pronoun heard. This proposition is further supported by findings that imply distance-neutral use (Larjavaara, 2007; Pajusalu, 2009) or spatial unmarkedness (Reile, 2019; Reile et al., 2019) of demonstrative pronoun *see*—“this/that” in the Estonian one-term system (NE), whereas in the Estonian two-term system (SE), demonstrative pronouns are both argued to be spatially anchored (Reile, 2019; Reile et al., 2020). Moreover, a similar tendency to first mark the region with a demonstrative adverb and then the referent with a demonstrative pronoun has been detected in the production of Estonian demonstratives (Reile, 2016) by the SE speakers as well as in the use of Finnish demonstratives by native Finnish speakers. For Finnish, a Finno-Ugric language with three demonstrative stems, Laury (1996) has argued that the locative demonstratives are used for referents that are conceptualized rather as a ground (i.e., a region) than a figure (i.e., the referred object), whereas demonstrative pronouns are used for figure-like referents.

Our additional finding that the SE speakers were more likely to choose a referent rather than opting for a “do not know” answer indicates that having more demonstratives in a demonstrative paradigm can help the speakers to handle ambiguous referential situations. This is in line with Diessel’s (1999) proposition that the more demonstratives a language has, the more aspects of the referent they can express. Although the Estonian demonstratives do not express visual salience of the referent *per se*, having an additional demonstrative pronoun seems to aid the speaker to reach a decision, i.e., to choose a referent.

Our fifth hypothesis stating that the RTs for choosing a referent, when the demonstrative *too—*“that” is heard, are slower for the NE speakers than for the SE speakers did not hold true. The origin of the participants proved to be significant only in the interaction with stimulus picture 4, where one of the referents was in near space and two were in far space. The tendency in the RTs seemed to be that the SE speakers were slower in choosing the referent than the NE speakers while hearing the input sentence *võta too klots siit—*“take that brick from here” and seeing picture 4 as compared to seeing picture 1, where the referents were all in far space. The SE speakers were quicker than the NE speakers while hearing the input sentence *võta see klots siit—*“take this brick from here” and seeing picture 4 as compared to seeing picture 1. All differences in the RTs were induced by stimulus pictures. This was especially pronounced when the participants heard the input sentences that included distal demonstratives (both determiner and adverb). The participants were quicker with almost all other pictures than with picture 1 (where all the referents were located in far space), and it was probably harder to make a choice based on this particular input sentence. It is also possible that the difference in the RTs between the two speaker groups is so subtle that the design of the experiment did not capture it. Using a button press instead of a computer mouse to measure the RTs of the participants would have given us more accurate results. Therefore, the fifth hypothesis could still hold true when using a different measuring technique and is worth further research.

Comparing the two systems in one language already suggests differences in conceptualization of space through language. Adding the evidence that has been reported for other languages suggests that different demonstrative systems can define the way speakers conceptualize space. When more tools are available, the speakers are provided with a more clearly carved up space. For example, demonstrative adverbs are not only used to reinforce the meaning of demonstrative pronouns, as has been suggested by Diessel (2013), but they can also indicate whether the referent is near the addressee or the speaker as in spoken Brazilian Portuguese (Meira and Guirardello-Damian, 2018) (note that demonstrative pronouns are not used for that purpose in spoken Brazilian Portuguese). This further shows the importance of studying demonstrative adverbs in addition to pronouns to better understand the mechanisms of the demonstrative systems.

In addition to the distance of the referent, we tested for the effects of visual salience on the interpretation of demonstratives. Previous research on Estonian demonstratives suggested that the effect of visual salience manifests itself in the word order of the referential utterance rather than in the choice of specific demonstratives (Reile, 2016, 2019), that is, if a referent is not visually salient, then in the word order of the referential utterance the demonstrative adverb precedes the referential noun phrase (that might include a demonstrative pronoun). We tested this in hypothesis 3, and the results show that the visual salience had no impact on which of the referents were chosen. Although this might be true for Estonian demonstratives, research on English demonstrative pronouns has shown that visual access has an effect on demonstrative choice (Coventry et al., 2014). Moreover, there are languages that even have a demonstrative that is specifically used for invisible referents (Diessel, 1999). Therefore, visual salience or access might be less strong of a factor and more language specific than the distance of the referent.

## Conclusion

To conclude, our study shows that distance has an effect on the interpretation of Estonian demonstratives, and at the same time, the effect of visual salience is overridden by the distance. In addition, the results suggest that there are differences on how the different demonstrative pronoun system users conceptualize space. When using the Estonian with an influence of the SE dialect, the NE speakers rely on demonstrative adverbs in interpreting the referential utterance that includes demonstrative pronoun and adverb combinations, whereas the SE speakers also take into account the semantics of demonstrative pronouns. This shows the importance of studying demonstrative pronouns and adverbs together when tackling the working mechanisms of demonstrative systems.

## Data Availability Statement

The raw data supporting the conclusions of this article will be made available by the authors, without undue reservation.

## Ethics Statement

Ethical review and approval was not required for the study on human participants in accordance with the local legislation and institutional requirements. Written informed consent for participation was not required for this study in accordance with the national legislation and the institutional requirements.

## Author Contributions

MR, KA, and NP designed the experiments. MR and KA conducted the experiments. KA analyzed the data. MR interpreted the data. The manuscript text was prepared by MR, NP, and KA. Figures and the tables were prepared by KA. All the authors reviewed the manuscript and approved the final version of the manuscript to be published.

## Conflict of Interest

The authors declare that the research was conducted in the absence of any commercial or financial relationships that could be construed as a potential conflict of interest.
